# Autonomic dysreflexia in a tetraplegic patient due to a blocked urethral catheter: spinal cord injury patients with lesions above T-6 require prompt treatment of an obstructed urinary catheter to prevent life-threatening complications of autonomic dysreflexia

**DOI:** 10.1186/1865-1380-5-6

**Published:** 2012-02-01

**Authors:** Subramanian Vaidyanathan, Bakul Soni, Tun Oo, Peter Hughes, Gurpreet Singh, Kamesh Pulya

**Affiliations:** 1 Regional Spinal Injuries Centre, Southport and Formby District General Hospital, Town Lane, Southport, PR8 6PN, UK; 2 Department of Radiology, Southport and Formby District General Hospital, Town Lane, Southport, PR8 6PN, UK; 3 Department of Urology, Southport and Formby District General Hospital, Town Lane, Southport, PR8 6PN, UK; 4 Department of Cardiology, Southport and Formby District General Hospital, Town Lane, Southport, PR8 6PN, UK

## Abstract

**Background:**

The Manchester Triage System is commonly used as the triage system in emergency departments of the UK. As per the Manchester Triage System, patients presenting with retention of urine to the accident and emergency department are categorized to yellow, which denotes that the ideal maximum time to first contact with a treating clinician will be 60 min. Cervical spinal cord injury patients, in whom urinary catheter is blocked, may develop suddenly headache, sweating, high blood pressure, cardiac dysrhythmia, convulsions, intracranial bleed, and acute neurogenic pulmonary oedema as a result of autonomic dysreflexia due to a distended bladder.

**Case presentation:**

A 46-year-old male with C-6 tetraplegia developed urinary retention because of a blocked catheter. He was seen immediately on arrival in the spinal injuries unit. The blocked catheter was removed and a new catheter was about to be inserted. Then this patient said that the ceiling lights were very bright and glaring. Five milligrams of Nifedipine was given sublingually. This patient started having fits involving his head, face, neck and shoulders with loss of consciousness. A 14-French silicone Foley catheter was inserted per urethra without any delay and 300 ml of clear urine was drained. This patient recovered consciousness within 5 min. Computed tomography of the brain revealed no focal cerebral or cerebellar abnormality. There was no intra-cranial haemorrhage.

**Conclusion:**

This case illustrates that spinal cord injury patients with lesion above T-6, who develop retention of urine because of a blocked catheter, may look apparently well, but these patients can develop suddenly life-threatening autonomic dysreflexia. Therefore, spinal cord injury patients, who present to the accident and emergency department or spinal units with a blocked urinary catheter, should be seen urgently although their vital signs may be stable on arrival. Increasing the awareness of staff in emergency departments regarding autonomic dysreflexia as well as education of the patient and carers will be useful in preventing this complication in persons with spinal cord injury.

## Background

Triage is a system of clinical risk management intended to ensure that patients are allocated to a clinical priority category depending upon the signs and symptoms. The Manchester Triage System has been shown to be a reliable system of triage in the emergency department [1]. According to the Manchester Triage System [2], patients are allocated to one of five categories (Table 1). General discriminators under each category are listed in Table 2. Specific discriminators for urinary problems are listed in Table 3. Patients presenting with retention of urine are categorized to yellow, which denotes that the ideal maximum time to first contact with a treating clinician will be 60 min.

**Table 1:** The Manchester Triage System [2]

Number	Name	Colour	Maximum waiting time (minutes)
1	Immediate	Red	0
2	Very urgent	Orange	10
3	Urgent	Yellow	60
4	Standard	Green	120
5	Non-urgent	Blue	240

**Table 2:** General Discriminators for each category as per the Manchester Triage System [2]

Patient category	General discriminators
Red	Airway compromise; inadequate breathing; exsanguinating haemorrhage; shock; currently fitting; unresponsive child
Orange	Severe pain; uncontrollable major haemorrhage; altered consciousness level; hot child; very hot adult
Yellow	Moderate pain; uncontrollable minor haemorrhage; history of unconsciousness; hot adult
Green	Recent mild pain; warmth recent
Blue	Recent mild pain; warmth recent

**Table 3:** Specific discriminators for urinary problems as per the Manchester Triage System [2]

Red	Airway compromise; inadequate breathing; exsanguinating haemorrhage; shock
Orange	Severe pain; priapism; hot child; very hot adult
Yellow	Colicky pain; frank haematuria; retention of urine; persistent vomiting; hot adult; moderate pain
Green	Recent mild pain; Vomiting; Dysuria; Recent urinary problem
Blue	Recent mild pain; vomiting; dysuria; recent urinary problem

Spinal cord injury patients with lesions above the T-6 level, in whom the urinary catheter is blocked, may develop suddenly headache, sweating, high blood pressure, convulsions, cardiac dysrhythmia, and intracranial bleed because of autonomic dysreflexia caused by a distended bladder. Autonomic dysreflexia might predispose a patient to atrial fibrillation by altering the pattern of repolarization of the atria, making the heart susceptible to a reentrant type of arrhythmia. High-level spinal cord injured patients may be at increased risk for the development of atrial fibrillation, an arrhythmia that, if left untreated, can increase the incidence of an embolic cerebrovascular accident that could further impair the patient's functional status [3,4]. Colachis and Clinchot gave an account of autonomic dysreflexia in a patient with C6 tetraplegia, who developed recurrent ventricular fibrillation and cardiac arrest [5]. Acute neurogenic pulmonary edema has been associated with episodes of autonomic dysreflexia and a patient with C-5 tetraplegia died following autonomic dysreflexia; autopsy findings showed gross and microscopic evidence of pulmonary edema [6].

We report a cervical spinal cord injury patient who presented with blockage of a urinary catheter. This patient developed generalised convulsions because of autonomic dysreflexia, which was caused by a distended urinary bladder. The Royal College of General Practitioners Centre for Commissioning [7] states "Prevention of an acute, avoidable episode is as important as service provision to address it". By adopting a strategy to see tetraplegic patients with blocked urinary catheters within 10 min, the risk of developing life-threatening autonomic dysreflexia will be reduced.

## Case Presentation

A 25-year-old British male dived into hotel swimming pool in 1989 and sustained a C-6 fracture. Anterior fusion of the cervical spine was performed with a bone graft taken from the right iliac crest. This patient required tracheostomy for clearance of secretions. Neurological examination revealed tetraplegia at C-6 (American Spinal Injury Association Grade A). He has been managing his bladder by an indwelling urethral catheter. The catheter was changed every 4 weeks by a health professional. This patient could not recollect developing autonomic dysreflexia in the past.

This patient rang the Spinal Injuries Centre on a Sunday in 2010 and informed the staff that his catheter was blocked. This patient was advised to come to the Spinal Injuries Centre immediately. This patient's brother brought him to the Spinal Unit within half an hour and he was attended to promptly. This patient looked well; he did not have sweating, headache, goose pimples or flushing of the face. He was alert and made the usual conversation about going to his favourite public houses for drinks. He was not breathless. He did not have increased spasms. This patient did not have to wait for any time. This patient's brother lifted him, put him on the bed and undressed him. The blocked catheter was removed. The external urethral meatus was cleaned with chlorhexidine prior to catheterisation. Then this patient, who was lying on the bed, said that the ceiling lights were very bright and glaring. Five milligrams of Nifedipine was given sublingually. This patient started having fits involving his head, face, neck and shoulders with loss of consciousness. A 14-French silicone Foley catheter was inserted per urethra without any delay and 300 ml of clear urine was drained. After this patient received Nifedipine, his blood pressure was 84/51 mmHg. A Venflon was inserted in his foot. Blood tests revealed: haemoglobin, 14.4 g/dl; white cell count, 11.3 × 10^9^/l; urea, 2.5 mmol/l; creatinine, 49 umol/l; glucose, 5.4 mmol/l.

This patient recovered consciousness within 5 min. Two hundred forty milligrams of Gentamicin was administered intravenously, as spinal cord injury patients with blocked catheters are susceptible to developing urine infections. This patient recovered well and he was able to take his tea. This patient was admitted to the Spinal Injuries Centre for observation.

A request was made for a brain scan for this patient. A blocked catheter and distended bladder led to autonomic dysreflexia and the patient developed convulsions. Computed tomography of the brain would show whether this patient had developed an intracranial bleed as a result of a transient hypertensive episode due to autonomic dysreflexia. CT of the brain revealed no focal cerebral or cerebellar abnormality. There was no intra-cranial haemorrhage.

In order to prevent recurrence of autonomic dysreflexia due to a blocked catheter, it was decided to take possible measures to prevent blockage of the urinary catheter. He was advised to drink plenty of fluids. This patient was requested to get his catheter changed more frequently. In case of blockage of the catheter, this patient was instructed to take Nifedipine 5 mg sublingually in order to prevent a rise in blood pressure due to autonomic dysreflexia. This patient was advised to carry Nifedipine capsules with him at all times. His carers were trained how to administer Nifedipine sublingually.

At present this patient was taking Oxybutynin 5 mg once a day. After discussing with him, this patient was prescribed 10 mg of modified-release Oxybutynin once a day. He was also prescribed an alpha-adrenoceptor blocking drug, Doxazosin modified-release 4 mg once a day. Doxazosin is likely to reduce the frequency and severity of autonomic dysreflexia. This patient was informed of the side effects of long-term indwelling catheter drainage. For example, long-term indwelling urinary catheters are often associated with problems such as urinary infection, blocked catheters and stones in the bladder. Intermittent catheterisation was preferable to long-term indwelling catheters. Unfortunately, this patient did not have carers who could perform intermittent catheterisations.

## Discussion

Autonomic dysreflexia is a potentially life-threatening episodic hypertension that develops in 50-90% of people with tetraplegia or high paraplegia. Autonomic dysreflexia occurs after spinal cord injury at or above the sixth thoracic spinal segment, because injury at this level leaves the sympathetic control of the extensive abdominal circulation amenable to unrestrained spinal reflexes. Mechanisms for autonomic dysreflexia that have been considered include upregulation of vascular catecholamine receptors, increased neural release of catecholamines, decreased pre-synaptic reuptake of catecholamines, loss of the baroreceptor reflex, altered glutamatergic control of spinal neurons and loss of tonic bulbo-spinal inhibitory input to spinal neurons [8]. Gao and associates [9] found significant sympathetic firing during autonomic dysreflexia that could couple with enhanced catecholaminergic vasoconstriction to cause large increases in arterial pressure. During autonomic dysreflexia, increases in arterial pressure are induced by sensory input entering the spinal cord below the level of the lesion causing debilitating headaches, sweating, seizures, strokes and even death. This hypertension can be initiated by routine daily procedures such as bladder distension, catheterization and bowel evacuation. Typically, the headache attributed to autonomic dysreflexia is a sudden-onset, severe headache accompanied by several signs and symptoms including increased blood pressure, altered heart rate, and diaphoresis cranial to the level of spinal cord injury, which is triggered by different noxious and non-noxious stimuli [10].

Jackson and Acland [11] found that 29 of 70 staff in the emergency department could not answer any questions on autonomic dysreflexia. The emergency department staff scored an average mark of 2 out of a possible 29. These researchers recommended that because of the potentially serious complications of autonomic dysreflexia, staff in the emergency department require teaching on autonomic dysreflexia accompanied by permanent reminders in the form of posters. This case is a reminder to health professionals working in emergency departments and spinal injury units that tetraplegic patients with blocked urinary catheters should be attended to promptly, as these patients are susceptible to develop life-threatening autonomic dysreflexia.

Yoo and associates [12] described a 45-year-old quadriplegic male who suffered left basal ganglia and thalamic haemorrhage associated with autonomic dysreflexia. These authors stated that a *preventive measure rather than episodic treatment of autonomic dysreflexia may be of paramount importance to avoid life-threatening complications of autonomic dysreflexia*. We also wish to emphasize the preventive aspects of autonomic dysreflexia in persons with spinal cord injury. The aim of this case report is to raise awareness of the possibility of a spinal cord injury patient developing autonomic dysreflexia as a result of a blocked urinary catheter among health professionals working in the emergency department and spinal injuries units. By relieving urinary retention promptly in these patients, the chances of occurrence of autonomic dysreflexia will be reduced greatly.

Education of the patient, carers and family members regarding autonomic dysreflexia is vital in order to prevent autonomic dysreflexia and to recognize its occurrence without delay. McGillivray and associates [13] highlighted the importance of promoting knowledge about recognising and managing autonomic dysreflexia. These authors stated that there was also a need to identify effective strategies for translating knowledge about autonomic dysreflexia and to ensure that these strategies were effective for persons of different ages and with different learning styles. Early recognition and prompt treatment of this life-threatening, but easily recognised condition is imperative to minimize effects on a patient's health status. In turn, the prevention and effective management of autonomic dysreflexia may ultimately reduce the risk of cardiac and cerebrovascular death in this population.

Spinal cord injury patients, who are susceptible to developing autonomic dysreflexia, should preferably carry an "Alert Card" with them. When a patient develops symptoms of autonomic dysreflexia such as headache, sweating, and goose pimples, the following basic systematic approach should be followed.

Sit upright if in bed (helps to lower blood pressure)

Blood pressure monitored every 3-5 min in the hospital

Loosen tight clothing and binders

Assess for cause - bladder and bowel first

Prepare for catheter change or bowel care

Find cause: bladder

Check urine in drainage bag

Full, empty and monitor

If empty, when last emptied

Check for kinks in tubing

Check for signs if catheter is dislodged

Any sign of haematuria?

Catheter blocked? Change it

Find cause: bowel

When did the patient have last bowel care?

Result - good/small

Lie on left side (head raised)

Per rectal check

If full will need manual evacuation

Local anaesthetic gel (3 min)

Gentle manual evacuation (ME)

Let autonomic dysreflexia resolve and then perform usual bowel care

Other cause?

?Ingrown toe nail

?Pressure ulcer

Give analgesia

Give Nifedipine 5-10 mg sublingually or glyceryl trinitrate 300 μg/nasal spray (only if diastolic blood pressure is greater than 100 mmHg) Patient should always have a supply of Nifedipine capsules for an emergency

## Conclusion

This case illustrates that spinal cord injury patients with a lesion above T-6, who develop retention of urine because of a blocked catheter, may look apparently well, but these patients can suddenly develop life-threatening autonomic dysreflexia manifested by pounding headache, high blood pressure, cardiac dysrhythmia, convulsions, intra-cranial bleed, and acute neurogenic pulmonary oedema. Therefore, spinal cord injury patients with a lesion above T-6 who present to the accident and emergency department or spinal injuries units with a blocked urinary catheter should be seen urgently although their vital signs may be stable on arrival.

## Consent

Written informed consent was obtained from the patient for publication of this case report and accompanying images. A copy of the written consent is available for review by the Editor-in-Chief of this journal.

## Competing interests

The authors declare that they have no competing interests.

## Authors' contributions

SV conceived the idea and wrote the manuscript. PH reported the medical images. BMS was the consultant in charge of the patient. All authors participated in providing care to this patient. All authors read and approved the final manuscript.
